# Before Hyperemesis Gravidarum Is Blamed for Thiamine Deficiency, Alternative Causes Should Be Ruled Out

**DOI:** 10.5811/cpcem.66390

**Published:** 2026-10-20

**Authors:** Josef Finsterer

**Affiliations:** Neurology & Neurophysiology Center, Vienna, Austria

Dear Editor:

We read with interest the article by Kreutzer et al about a 22-year-old pregnant woman who developed Wernicke encephalopathy (WE) in the context of hyperemesis gravidarum at 13 weeks of gestation.^1^ Thiamine deficiency was detected and attributed to the hyperemesis gravidarum.^1^ The WE resolved completely after thiamine supplementation.^1^ The study is promising, but some points should be discussed.

First, we disagree with the statement in the highlights that only a single case of hyperemesis gravidarum and WE has been described to date.^1^ In addition to the case described, WE in association with hyperemesis gravidarum has also been reported by Kotha et al,^2^ Kantor et al,^3^ Bensaid et al,^4^ and many other authors. A PubMed search using the search terms “WE” and “hyperemesis gravidarum” yielded over 200 results. These findings do not explain why only a single case of WE due to hyperemesis gravidarum was identified.

Second, the patient’s usual diet was not documented.^1^ Was there any evidence of malnutrition or alcohol dependence? Thiamine deficiency is particularly common in patients who consume refined carbohydrates such as polished white rice, white flour, and white sugar, because the outer bran and germ layers of grains are rich in thiamine. A diet high in carbohydrates and low in protein can also cause thiamine deficiency. Similarly, severely malnourished individuals and those with eating disorders are known to often have insufficient thiamine intake. Furthermore, foods high in freshwater fish, fresh shellfish, or certain ferns can degrade and destroy thiamine because they are rich in thiaminases.

Third, the patient’s current medications (cefdinir, doxylamine, ondansetron, pyridoxine, and loratadine) were not considered as a possible cause of the confusion. Ondansetron, in particular, is known to induce confusion.^5^ Furthermore, it is conceivable that at least cefdinir, or the combination of the four substances, impaired intestinal thiamine absorption, although these medications are not known to directly cause thiamine deficiency. We should also know which medications the patient took for hyperemesis gravidarum in the days prior to admission.

The fourth point is that the duration of the vomiting was not documented.^1^ Since the patient was discharged after only five days, it is very likely that it was short-lived. Therefore, it is rather unlikely that hyperemesis gravidarum was actually responsible for the low thiamine level. Since the patient developed confusion during the five-day hospital stay, it cannot be ruled out that this was due to the urinary tract infection, its treatment, or the concurrent hyperemesis. Because thiamine deficiency can cause vomiting, it should be ruled out that the vomiting was already a manifestation of a pre-existing thiamine deficiency.

In summary, vomiting must be prolonged to be considered as a cause of thiamine deficiency. Before thiamine deficiency is considered as the cause of vomiting, other causes must be excluded.
